# Dosimetric implications of kidney anatomical volume changes in ^177^Lu-DOTATATE therapy

**DOI:** 10.1186/s40658-024-00672-w

**Published:** 2024-08-02

**Authors:** Jehangir Khan, Tobias Rydèn, Martijn Van Essen, Johanna Svensson, Joseph Grudzinski, Peter Bernhardt

**Affiliations:** 1 Department of Medical Physics and Biomedical Engineering (MFT), Sahlgrenska University Hospital, Gothenburg, SE-41345 Sweden; 2 Department of Medical Physics, Faculty of Medicine and Health, Örebro University Hospital, Örebro, Sweden; 3 Department of Clinical Physiology, Sahlgrenska University Hospital, Gothenburg, Sweden; 4 Department of Oncology, Institution of Clinical Sciences, Sahlgrenska Academy at University of Gothenburg, Gothenburg, Sweden; 5 Department of Medical Radiation Sciences, Institute of Clinical Sciences, Sahlgrenska Academy at University of Gothenburg, Gothenburg, USA; 6 Department of Radiology, University of Wisconsin – Madison, Madison, USA

**Keywords:** ^177^Lu-DOTATATE, Neuroendocrine, Single photon emission tomography, SPECT/CT, Kidney dosimetry, Recovery coefficient, Kidney parenchymal volume

## Abstract

**Introduction:**

This study aims to evaluate the use of CT-based whole kidney parenchyma (WKP) segmentation in ^177^Lu-DOTATATE dosimetry. Specifically, it investigates whether WKP volumes change during treatment and evaluates the accuracy of applying a single delineated WKP volume for dosimetry. Furthermore, it aims to determine the cause of WKP volume changes—whether caused by radiation or amino acid infusion—by comparing them with spleen volume changes as a marker for radiation-induced alterations.

**Methods:**

SPECT/CT images of 18 patients were acquired over the abdomen approximately 4 h (h) (D0), 24 h (D1), 48 h (D2) and 168 h (D7) post-administration of ^177^Lu-DOTATATE. CT guided WKP volumes were measured before (baseline) and during treatment. Kidney activity concentrations at each time point were derived from CT-segmented WKP overlaid on SPECT scans. The accuracy of using WKP segmentation from a single CT for all time points was assessed against the gold standard of segmenting each WKP individually. Time-integrated activity calculations were based on a tri-exponential curve fit of the kidney activity concentration over time. Kidney absorbed doses were estimated under the assumption of local energy deposition. Additionally, the impact of various partial volume correction methods on dosimetry was evaluated.

**Results:**

Whole-kidney parenchyma (WKP) volumes, ranging from 31 to 243 mL, showed a gradual increase from baseline (mean ± SD = 130.6 ± 46.1 mL) at the initial time points D0 (138.5 ± 44.7 mL) and D1 (139.4 ± 41.6 mL), followed by a slight decrease at D2 (132.8 ± 44.5 mL) and a further decrease at D7 (129.2 ± 42.7 mL). The volume increase at D0 and D1 was statistically significant. Spleen volume did not change during treatment, suggesting that amino acid infusion rather than irradiation effects caused WKP volume changes. Bland-Altman analysis revealed WKP volume biases of 8.77% (D0 vs. B_L_), 10.77% (D1 vs. B_L_), 1.10% (D2 vs. B_L_), and 1.10% (D7 vs. B_L_), with corresponding uncertainties of 24.4%, 23.6%, 25.4%, and 25.4%, respectively. When WKP segmentation from a single CT is applied across all SPECTs, these WKP volume changes could overestimate the activity concentration and mean absorbed doses up to 4.3% and 2.5%, respectively. The absorbed dose uncertainties using a recovery coefficient (RC) of 0.85 for single-time-point WKP delineation increase the absorbed dose uncertainty by 4% compared to the use of patient-specific RCs and time specific segmentation of WKP volumes.

**Conclusions:**

Kidney volume exhibited significant variation form D0 to D7, affecting the precision of dosimetry calculation, primarily due to errors in whole-kidney parenchyma (WKP) delineation. Notably, using WKP segmentation from a single CT scan applied to sequential SPECT images introduce further uncertainty and may lead to an overestimation of the absorbed dose. The fluctuations in kidney volume are most likely attributable to amino acid infusion.

## Introduction

Peptide receptor radionuclide therapy (PRRT) with ^177^Lu-DOTA-D-Phel-Tyr3-octreotate (^177^Lu-DOTATATE) shows favourable therapeutic effects on tumour growth in patients with somatostatin receptor positive neuroendocrine tumors (NETs) [1–3]. The NETTER-1 trial reported progression-free survival in midgut NETs with a response rate approaching 1 in 5 patients treated with four cycles of 7.4 GBq ^177^Lu-DOTATATE at an eight-week interval per cycle [4].

To maximize the treatment effectiveness of ^177^Lu-DOTATATE, the optimized amount of activity and the appropriate number of treatment cycles can be administered to NETs patients, which maximizes the absorbed dose in the tumors, without increasing the risk for clinically significant side effects on organs at risk (OAR), namely the kidney and bone marrow [5, 6]. Therefore, to support whether a patient should continue further treatment with ^177^Lu-DOTATATE, individual dosimetry after each cycle of treatment is necessary to evaluate the cumulative absorbed dose to the OAR.

In PRRT, a generally accepted kidney absorbed dose limit of 23 Gy is adopted from external beam radiotherapy (EBRT) [7]. However, studies [8, 9] have shown that higher kidney absorbed dose limits (28–29 Gy), even increased to 40 Gy [10, 11] for patients without risk factors, could potentially be adopted for PRRT due to the lower dose rate and more inhomogeneous absorbed dose distribution compared with EBRT. Another difference between EBRT and PRRT dosimetry is the magnitude of uncertainty, which is generally less than 5% for EBRT but considerably greater than 10% for PRRT. The major sources of uncertainty in kidney dosimetry have been described [12]. In the order of decreasing magnitude, they are quantitative SPECT measurements ~ 12%, kidney delineation and partial volume correction ~ 10%, and uncertainties related to dose calibrator and activity measurements ~ 5%. However, the change in kidney volume during treatment has not been evaluated which is another factor that may influence the kidney dosimetry accuracy.

In a study by Sandström et al., [13] it was noted that a SPECT based threshold segmentation technique resulted in significantly different functional kidney volumes between day one (D1) and seven (D7) after administration of ^177^Lu-DOTATATE. The cause of anatomical kidney volume variations and it influence on kidney dosimetry were not evaluated.

We speculate that kidney volume change might be due to the infusion of the amino acids, used for reducing the kidney uptake of ^177^Lu-DOTATATE, and that changes in kidney volume during treatment might influence kidney dosimetry.

The filtration of the radiolabeled peptides occurs in the renal glomerulus following interaction with anionic charges on cell membranes, radioligands or their radiometal chelate. As a result, the metabolic products are trapped in the proximal tubular cells, where they become a radiation source for the nearby highly radiosensitive glomeruli [14, 15]. Infusion of the amino acids lysine and arginine provides renal cytoprotection by blocking renal re-uptake of ^177^Lu-DOTATATE peptide fragments [16], allowing the administration of larger amounts of radioactivity with a similar safety margin. The infusion of amino acids is considered safe [17]. Nevertheless, the large volume of 1000 to 2000 mL of amino acid solutions might influence kidney volume during treatment, due to impairments of protein reabsorption and maintenance of anion balance of the renal tubule system. Hence, amino acid infusion can temporarily increase kidney volume due to enhanced blood flow and glomerular filtration rate (GFR), causing tubular enlargement from osmotic diuresis. Although, glomeruli may initially experience an increased filtration load, this typically return to normal thereafter.

Kidney dosimetry in radionuclide therapy relies on the accurate quantification of radioactivity over time. This requires precisely delineating WKP volumes on the acquired CT images at several time points’ post-treatment and the appropriate correction of partial volume effects (PVEs), which can vary with kidney volume and shape. In this study, simulated patient-specific RCs were used for partial volume correction for the estimation of kidney-absorbed doses as described in previous study [18].

The aim of the present work was to measure the change in kidney volume throughout the course of ^177^Lu-DOTATATE treatment and to estimate the dosimetric uncertainties associated with the kidney volume change. Based on our results, we then provide recommendations for dosimetry workflows.

## Materials and methods

Eighteen patients diagnosed with progressive metastatic NETs and treated with [^177^Lu] Lu-DOTATATE (Lutathera^®^) at the Sahlgrenska University Hospital between 2019 and 2021 were included in this study. This study was approved by the regional Ethics Review Board in Gothenburg, waiving the requirement of consent to participate (2020–05092), and was performed in accordance with the declaration of Helsinki and national regulations. Table 1 describes the patient characteristics.

**Table 1:** Patient characteristics summary. Numerical variables are given as average (range) and categorical variables as N in the data, respectively

Characteristics	Data
Sex, N	Female: 10 Male: 8
Age (y), average (range)	71 (56–86)
Site of primary tumour, N (%)	Small intestine: 10 (56%) Unknown: 8 (44%)
Body surface area (m^2^), median (range)	1.84 (1.45–2.29)
eGFR (ml/min/1.72 m^2^), median (range)	74 (51–88)
Administered activity (GBq), median (range)	7.5 (7.4–7.6)
SPECT/CT scanning times post injection (hours), median (range))	D0: 4.1 (1.3–5.8) D1: 22.3 (19.5–24.5) D2: 49.3 (43.8–51.2) D7: 171.1 (169.1–173)

### ^177^Lu-DOTATATE administration

Each patient was administered an intravenous infusion of kidney-protective amino acid solutions (Vamin 14 gN/l, 2000 mL, Fresenius Kabi AG, Bad Homburg, Germany). Administration of the amino acid solutions started 25–30 min prior to ^177^Lu- DOTATATE with an infusion speed of 400 mL/h. A total of 2 L of amino acid solutions were administered to each patient. The product vial was measured before and after the treatment in a radionuclide calibrator (VIK-202 5051, Comecer, SN: 21605-5051-07) to compute the net administered activity. The net administrated ^177^Lu-DOTATATE activity for this cohort was 7.5 GBq (range, 7.4 − 7.6).

### Image acquisition

The SPECT/CT acquisitions were acquired approximately 4 hours (h) (D0), 24 h (D1), 48 h (D2) and 168 h (D7) after ^177^Lu-DOTATATE injection (Table 1). Imaging was performed using a SPECT/CT system, Discovery NM/CT 670 (GE Healthcare, Waukesha, WI, USA). The matrix size of the CT was 512 × 512 with a pixel size of 0.98 mm and a slice thickness of 5 mm. The gamma camera was equipped with a NaI (Tl) crystal thickness of 5/8”, medium-energy parallel- hole collimator, and the energy window was set at 208 keV ± 10%. SPECT imaging was performed over the upper abdomen including the kidneys, liver, and spleen. The SPECT images were acquired with a 30s frame duration for 120 projections. Monte Carlo-based ordered subset expectation maximization reconstruction (MC-OSEM) [19] was used for attenuated, scatter and collimator detector response corrected SPECT reconstruction. The matrix size was 128 × 128 with a pixel size and a slice thickness of 4.42 mm.

### Kidney delineation and activity quantification

The spleen and the whole kidney parenchymal (WKP) volume, including the renal cortex and medulla, and excluding the renal pelvis, were manually delineated on CT images for all time points, i.e., WKP_D0_, WKP_D1_, WKP_D2_, and WKP_D7_ (Table 1) after ^177^Lu-DOTATATE administration. The baseline WKP and spleen volumes (B_L_) were delineated from the CT in diagnostic PET/CT protocol; images acquired within 5 months before ^177^Lu-DOTATATE treatment. The CT was performed with a tube voltage of 120 KV, rotation time 0.5 s, pitch 0.8, and activated dose modulation function. The CT guided WKP volumes acquired after treatment were manually adjusted to fit the position of the left and right kidneys on corresponding SPECT, and SPECT scans obtained at different time points, which might differ from CT due to organ movement and misregistration of hybrid images [20]. Thus, the activity concentrations and volume (mL) were determined in the manually delineated kidney VOIs.

**Fig. 1:**
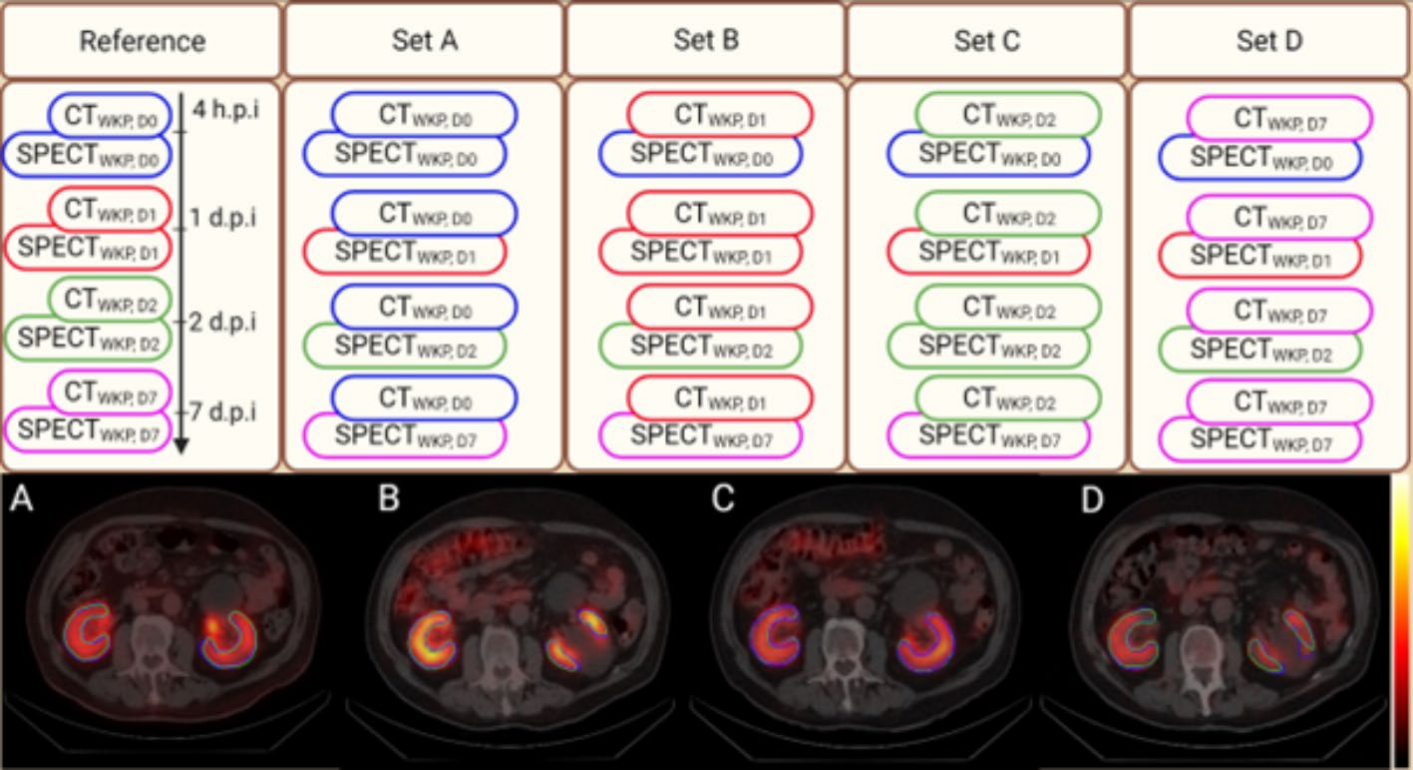
Top panel: This flowchart outlines the procedure for measuring activity concentrations using CT-guided whole kidney parenchyma (WKP) volumes, denoted as CT_WKP, D0_ (bue), CT_WKP, D1_ (red), CT_WKP, D2_ (green), and CT_WKP, D7_ (purple), each overlaid on corresponding SPECT images (reference). It also illustrates the application of a single CT-guided WKP volume from D0, D1, D2, and D7 across all time points, labeled as set A, set B, set C, and set D, respectively. Bottom panel: Panels A-D show the propagation of a single CT-segmented WKP volume of interest (VOIs) across all other time points on SPECT/CT images compared to their respective ground truth

The reference method (WKP_ref_) consisted on CT guided WKP acquired at all-time points, to determine the partial volume corrected activity concentrations on corresponding serial SPECT scans obtained after administration of ^177^Lu-DOTATATE. Further, this method was compared with methods where only one of the CT images were segmented (i.e., WKP_D0_, WKP_D1_, WKP_D2_, or WKP_D7_), and used for determining the partial volume corrected activity concentrations on serial SPECTs, and the WKP based kidneys absorbed doses. Moreover, WKP defined on single time-point CT were adjusted on serial SPECT without any geometry modification of VOIs (Fig. 1).

The time-integrated activity concentration (TIAC) was computed from the time activity curves obtained from a tri-exponential (Eq. 1) curve fit (with five parameters) to the four determined activity concentrations at D0, D1, D2, and D7, with an additional activity concentration equal to zero at the time where administration of ^177^Lu-DOTATATE started, and then integrated from zero time to infinity.

1 $$\:TIAC=\underset{0}{\overset{\infty\:}{\int\:}}({A}_{1}*{e}^{\left(-b*t\right)}+{A}_{2}*{e}^{\left(-c*t\right)}-{({A}_{1}+A}_{2})*{e}^{\left(-d*t\right)})dt$$

Where A1, A2, b, c and d are the fitting parameters and t is the time after administration of ^177^Lu-DOTATATE. The curve fit was performed in MATLAB (The MathWorks, Inc.; Natick, MA, USA).

The kidney self-absorbed doses (D) were calculated according the EANM guidelines using a kidney density of 1.05 g/mL, for converting the TIAC unit of decay/mL to decay/g, assuming local energy deposition (LED) of 147.9 keV per decay and multiplying it with a kidney self-absorption factor for including self-absorbed photon irradiation. This energy deposition was multiplied with TIAC and converted to the unit Gy:

2 $$\:D=\text{T}\text{I}\text{A}\text{C}\:.\:\text{L}\text{E}\text{D}$$

### Statistical analysis

Statistical analysis was performed in MATLAB (The MathWorks, Inc.; Natick, MA, USA). The Bland-Altman plots were used to analyze the agreement of the WKP volumes and the absorbed dose estimate comparing the reference method (WKP_ref_) with methods where only one of the segmented WKP volume i.e., WKP_D0_, WKP_D1_, WKP_D2_, and WKP_D7_ were used on serial SPECT scans. A nonparametric Wilcoxon paired test was performed to examine the significance difference in the WKP volumes defined on CT images among all time points; the activity concentrations measured in WKP methods in sequential SPECTs; and the kidneys absorbed doses estimate using reference method WKP_ref_ versus a single time point based CT (i.e., either WKP_D0_, WKP_D1_, WKP_D2_, or WKP_D7_) methods, respectively. A *P*-value less than 0.05 indicates a statistically significant difference.

## Results

**Fig. 2:**
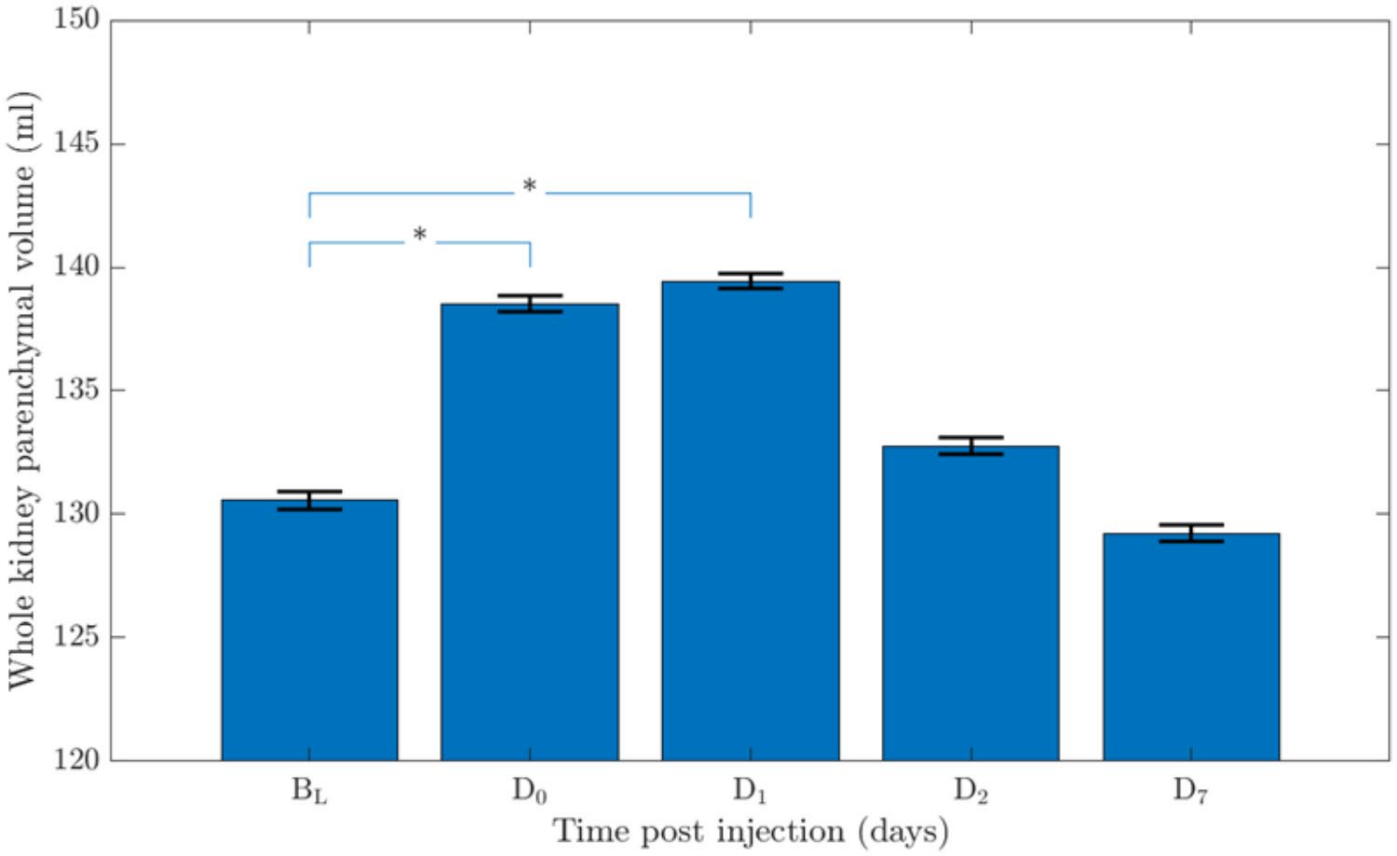
The whole kidney parenchyma volumes measured on diagnostic CT before (baseline volume B_L_) and during ^177^Lu-DOTATATE treatment at D0, D1, D2, and D7 post injection. The blue bars represent the mean and the black bars the standard error of the mean, respectively. Statistically significant differences are indicated with *, representing *p* ≤ 0.05

**Fig. 3:**
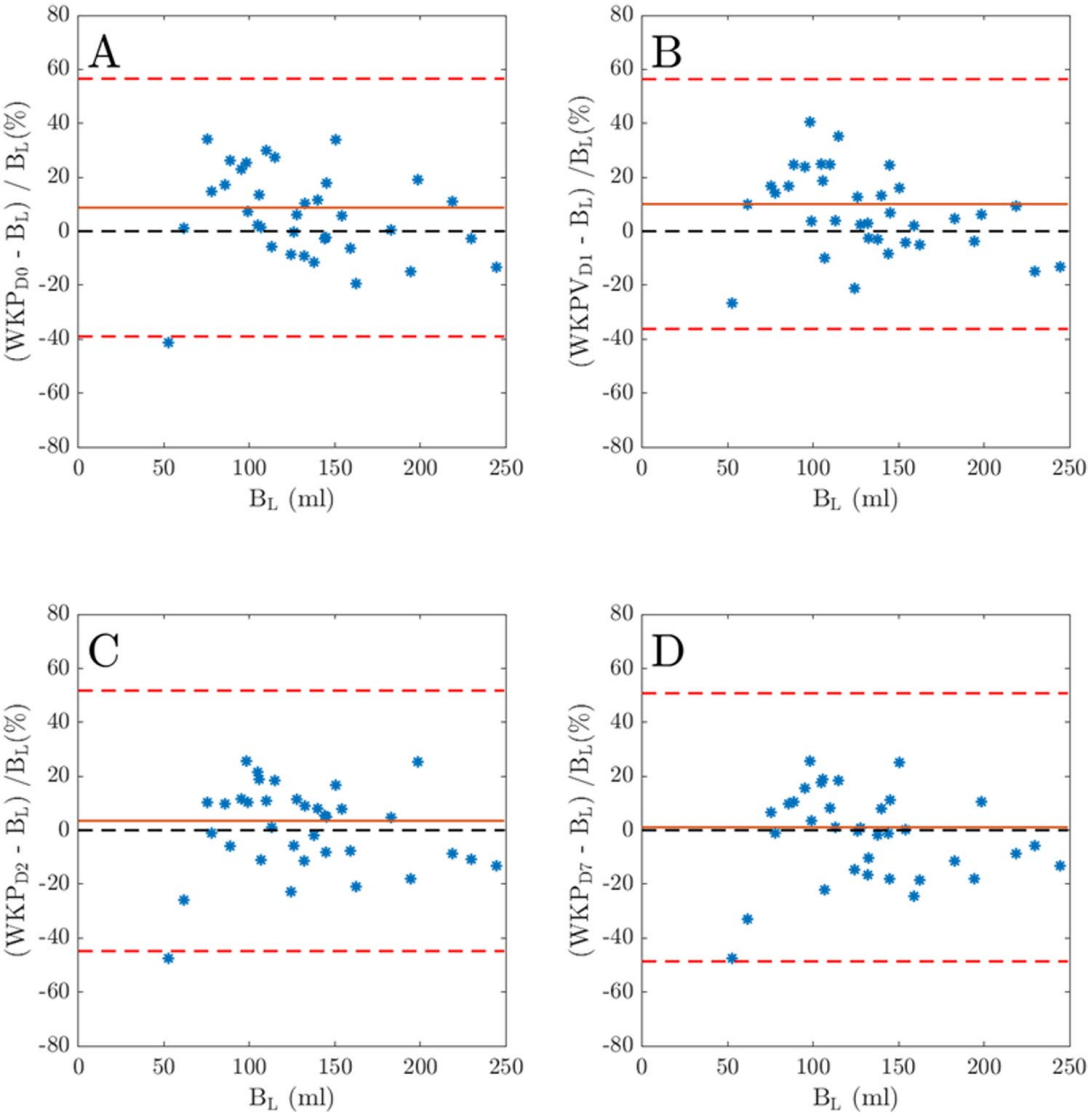
Bland-Altman plots comparing whole kidney parenchyma (WKP) volume during ^177^Lu-DOTATATE volumes with baseline WKP volume (B_L_). Panel **A**, **B**, **C** and **D** shows the plot for the WKP volumes at D0, D1, D2, and D7, respectively. The bias is shown with a solid red line, and the dashed red lines show the limits of agreement (± 1.96 SD). The dashed black line shows the line of agreement

The WKP volumes of the 180 (5 × 36 i.e. including B_L_volumes) manually delineated right and left kidneys were between 27.7 and 248.8 mL. When analyzing the individual WKP volumes over time after administration of ^177^Lu-DOTATATE, it was statistically confirmed that the WKP volumes changed over time. The largest volumes were obtained at D1 after the administration of ^177^Lu-DOTATATE. The mean (SD) of the WKP volumes before treatment B_L_ (i.e., baseline WKP volumes) and after treatmet measured at D0, D1, D2, and D7 were 130.6 (46.1), 138.52 (44.7), 139.4 (41.6), 132.8 (44.5), and 129.2 (42.7) mL, respectively (Fig. 2). There was a statistically significant difference in right and left WKP volumes at B_L_ versus D0 (*P* = 0.047), B_L_ versus D1 (*P* = 0.02), B_L_ versus D2 (*P* = 0.74), and B_L_ versus D7 (*P* = 0.55), respectively (Fig. 2). The Bland-Altman plots represents the relative WKP volume differences for the times D0, D1, D2 and D7 versus B_L_ (Fig. 3A–D). The bias and the standard deviation (SD) for the corresponding comparison were 8.77% (24.4%), 10.07% (23.6%), 1.10% (25.4%), and 1.10% (25.4%), respectively. The mean (SD) of the spleen volumes before treatment B_L_ (i.e., baseline spleen volumes) and after treatmet measured at D0, D1, D2, and D7 were 183.5 (115.1), 193.8 (134.8), 182.3(129.2), 193.7 (152.7), and 179.5 (116) mL, respectively. It was no statistically significant difference in spleen volumes acquired at B_L_ versus D0 (*P* = 0.39), B_L_ versus D1 (*P* = 0.89), B_L_ versus D2 (*P* = 0.72), and B_L_ versus D7 (*P* = 0.93), respectively.

### Influence of kidney segmentation on the activity concentrations

**Fig. 4:**
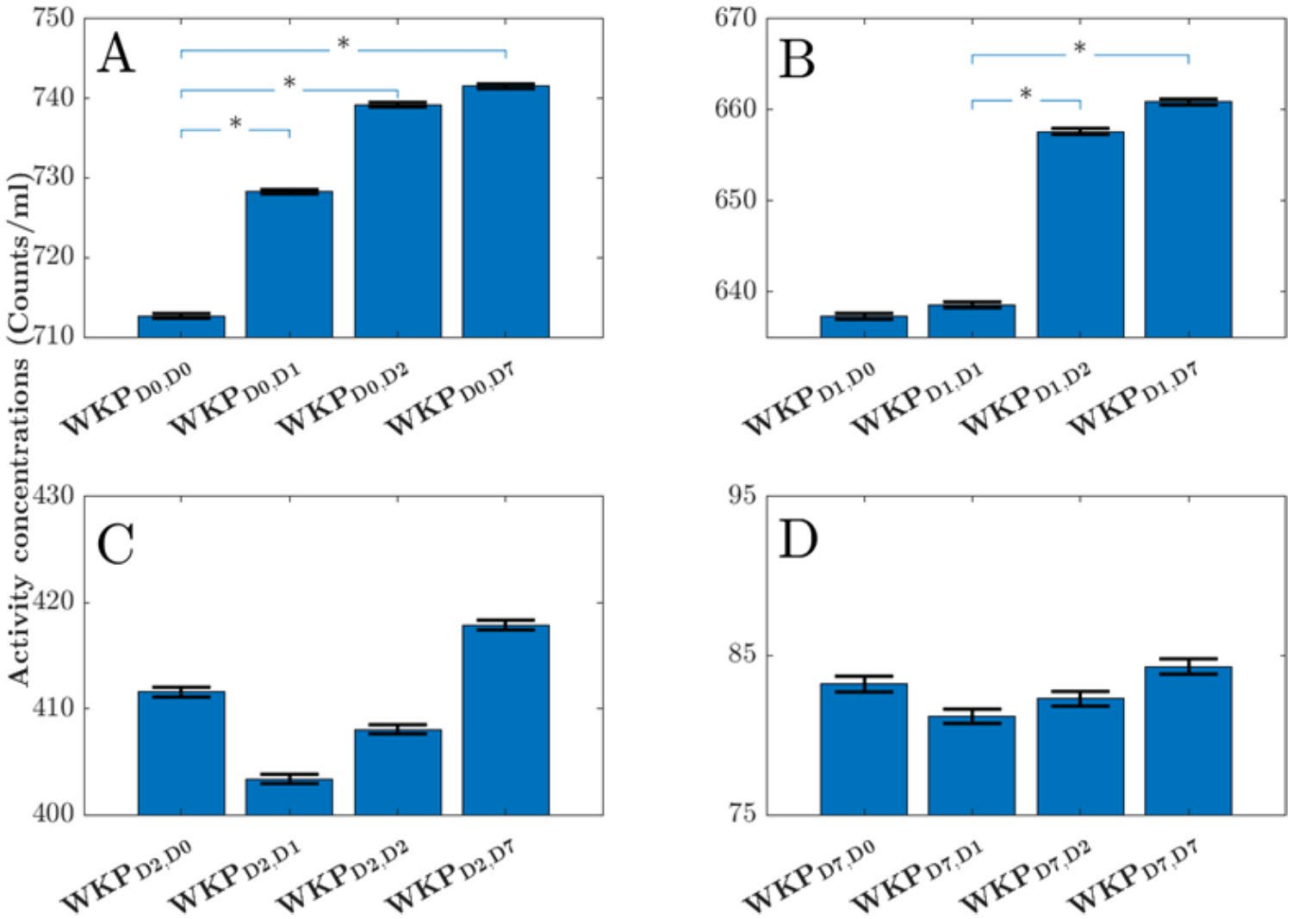
The mean (blue bars) and the standard error of the mean (black bars) of the whole kidney parenchyma (WKP) activity concentrations when using the WKP volume delineated in CT acquired at D0, D1, D2 and D3; panel **A**, **B**, **C** and **D** respectively. The index j in WKP_Di, Dj_ represents the time point the WKP volume was delineated, and index i is the time point for the measurement of the activity concentration in WKP. Statistical significant differences are indicated with * representing *p* ≤ 0.05

Figure 4. presents the mean kidney activity concentrations determined using different delineated whole kidney parenchyma (WKP) volumes. The activity concentrations at D0 (Fig. 4A) and D1 (Fig. 4B) were influenced by the specific segmented WKP volume of interest (VOI) used. However, at D2 (Fig. 4C) and D7 (Fig. 4D), the activity concentrations were not affected by the choice of segmented WKP volume.

Table 2 shows the results of the Bland-Altman analysis, indicating that using a single delineated WKP volume (whether it is WKPD0, WKPD1, WKPD2, or WKPD7) can lead to an overestimation of activity concentration by 2.7–4.3% at D0 and D1.

**Table 2:** The bias (SD) of the activity concentrations measured in SPECTs based on WKP volume delineated in CT scans at different time points. The bolded values indicate statistical significant differences

WKP_D0,D0_ (%) 0.0 (0.0)	**WKP**_**D0,D1**_ **(%)** **2.7 (4.6)**	**WKP**_**D0,D2**_ **(%)** **3.9 (6.7)**	**WKP**_**D0,D7**_ **(%)** **4.3 (4.9)**
WKP_D1,D0_ (%) 0.4 (5.9)	WKP_D1,D1_ (%) 0.0 (0.0)	**WKP**_**D1,D2**_ **(%)** **3.0 (5.7)**	**WKP**_**D1,D7**_ **(%)** **3.5 (5.5)**
WKP_D2,D0_ (%) -0.8 (5.4)	WKP_D2,D1_ (%) 0.8 (6.9)	WKP_D2,D2_ (%) 0.0 (0.0)	WKP_D2,D7_ (%) 2.2 (5.9)
WKP_D7,D0_ (%) -0.7 (7.3)	WKP_D7,D1_ (%) 0.2 (6.6)	WKP_D7,D2_ (%) 1.8 (7.4)	WKP_D7,D7_ (%) 0.0 (0.0)

### Influence of kidney segmentation on absorbed doses

**Fig 5.**
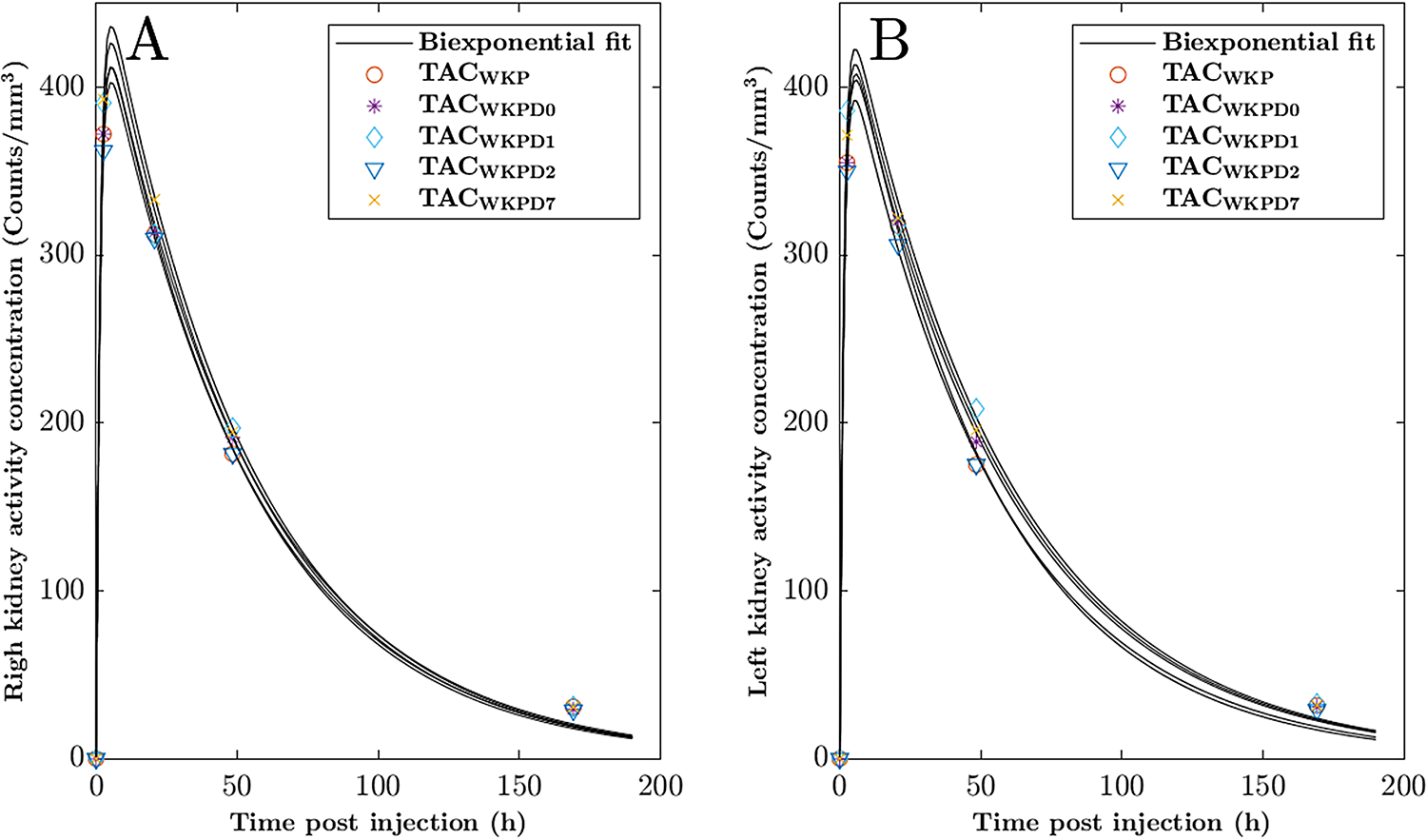
Illustration of five-time activity curves (TAC) for the right (**A**) and left (**B**) kidneys, respectively. The black lines represent the curve fitted data. The reference TACs, where the whole kidney parenchyma (WKP) was delineated in each CT at D0, D1, D2 and D7 for activity concentration measurement (TAC_WKP_), are indicated with circle (). The TACs for the simplified method with one delineated WKP volume are indicated with: WKP_D0_ (), WKP_D1_ (), WKP_D2_ (), or WKP_D7_ () and solid black lines, respectively

The time activity curves (TAC) of a patient with typical biokinetic behavior in the right (Fig. 5A) and left (Fig. 5B) kidney, respectively.

**Fig. 6:**
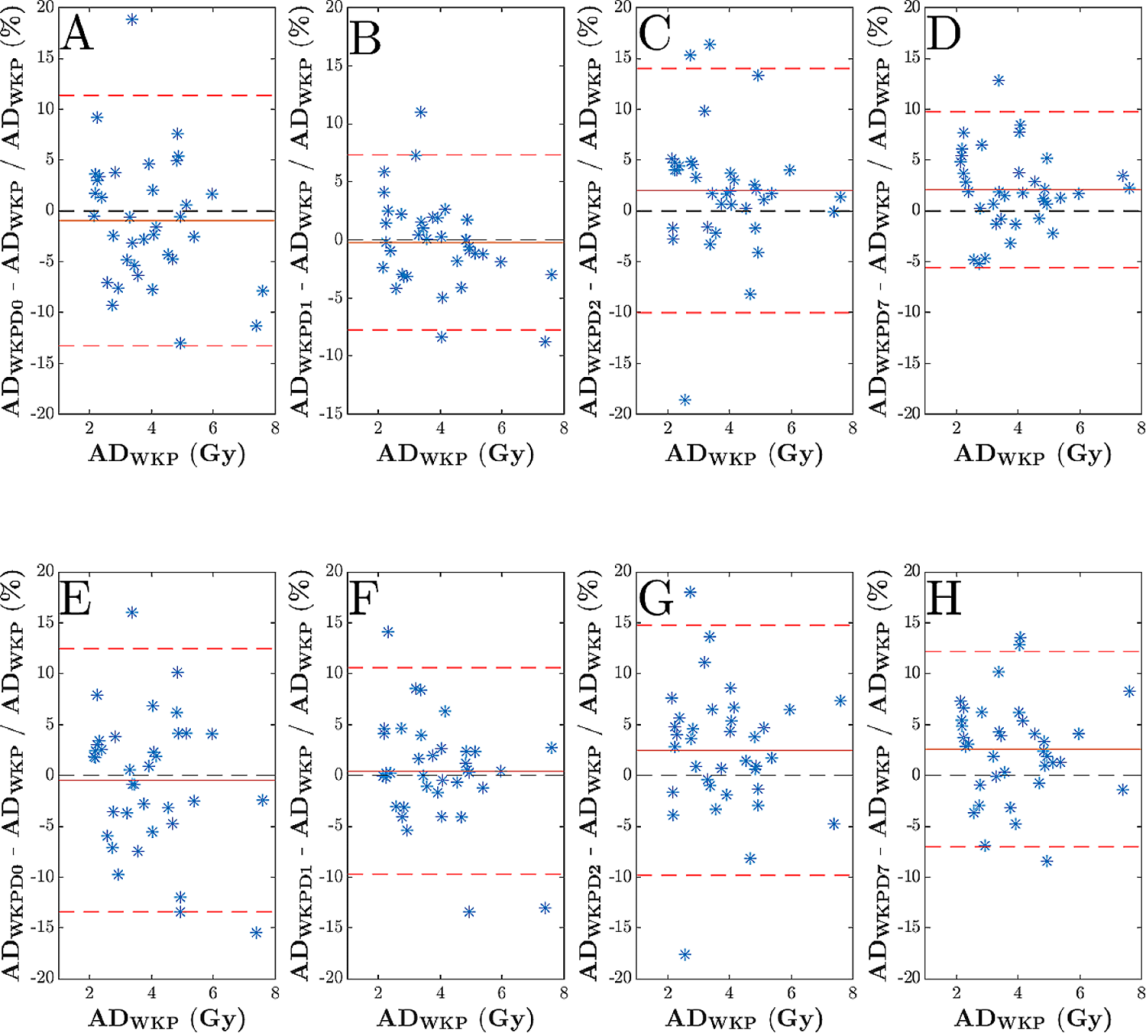
The Bland-Altman analysis of kidney absorbed doses (Gy) estimated from manually delineated whole kidney volumes when corrected with a recovery coefficient of 0.85 for the methods: (**A**) AD_WKPD0_, (**B**) AD_WKPD1_, (**C**) AD_WKPD2_, and (**D**) AD_WKPD7_, and with a specific RC for the methods (**E**) AD_WKPD0_, (**F**) AD_WKPD1_, (**G**) AD_WKPD2_, and (**H**) AD_WKPD7_, respectively. The solid red lines indicate the bias value while the dashed red lines indicate the line of alignment (± 1.96 SD). The dashed black lines indicate the line of identity

The mean (SD) of the estimated kidney absorbed doses for reference method (AD_WKP_) and the use of a single WKP VOI i.e., AD_WKPD0_, AD_WKPD1_, AD_WKPD2_, and AD_WKPD7_ with fixed RC equal to 0.85 were: 3.86 Gy (1.37), 3.80 Gy (1.28), 3.83 Gy (1.29), 3.93 Gy (1.39) and 3.93 Gy (1.40). The corresponding bias (SD) from the Bland-Altman plots (Fig. 6A and H) were − 0.49% (6.60%), 0.43% (5.18%), 2.46% (6.28%) and 2.58% (4.90%), respectively. A statistical significant difference was obtained against the reference method for AD_WKPD7_ (*P* = 0.0131). When patient-specific RCs were used, the mean (SD) kidney absorbed doses for AD_WKP_, AD_WKPD0_, AD_WKPD1_, AD_WKPD2_ and AD_WKPD7_ were 3.88 (1.39), 3.81 (1.30), 3.85 (1.30), 3.95 (1.40), and 3.95 (1.42). The corresponding bias (SD) from the Bland-Altman analysis were − 0.95% (6.29%), -0.21% (3.85%), 2.01% (6.13%), and 2.09% (3.91%), respectively. For statistical significance, the difference against the reference method was obtained for AD_WKPD2_ (*P* = 0.008) and AD_WKPD7_ (*P* = 0.003). In addition, the mean (SD) kidney absorbed doses for AD_WKP_ with patient-specific and 0.85 RC were 3.86 (1.39) and 3.88 (1.41), respectively. The bias (SD) from the Bland-Altman analysis were 0.43% (4.00%).

## Discussion

Our results demonstrate a statistically significant change in WKP during treatment with ^177^Lu-DOTATATE. The WKP at 4 h post injection (h.p.i.) and 24 h.p.i. was 9 and 11%, larger than the WKP at baseline and at 7 d.p.i. Although the intravenous administration of amino-acids causes enlargement of the WKP, it ultimately improves the effect of PRRT with ^177^Lu-DOTATATE therapy by allowing the administration of higher amounts of radiolabeled somatostatin analogue by protecting the kidneys. However, the excess of amino acids floods the renal tubule system [17]. This might be one explanation for the changes in kidney volume observed over time. In addition, non-significant variations in spleen volume observed in NETs patient during ^177^Lu-DOTATATE treatment, indicating that WKP volume changes are due to amino acid solution injection and not caused by irradiation effects. Our identification of change in WKP volume is consistent with previously published work on measurement of the change in functional volume by a threshold segmentation approach in SPECT [13].

Manual delineation of the kidneys is time consuming; therefore, one alternative approach is to segment WKP on one-time point CT image and propagate the contour to all other time points. However, such an approach does not account for volume changes during therapy. Our results demonstrate up to more than 4% overestimation of activity concentrations when the WKP is delineated at day 2 or day 7 after ^177^Lu-DOTATATE administration. The use of these single WKP for dosimetry will lead to an overestimation of the mean absorbed doses up to 2.5%. For the WKP delineated at 4–24 h.p.i, no statistical differences in absorbed doses were noted compared with the reference method. Consequently, these WKP are recommended for use in kidney dosimetry when amino acids is used for kidney protection in radionuclide therapy. In addition, it might be appealing to use delineated WKP from higher resolution imaging modalities such as MRI or diagnostic CT. Consequently, WKP imported from other modalities should be used with caution when the WKP volume may change during treatments. Sandström et al., [13] explored the impact of SPECT threshold segmentation on kidney dose estimates, observing significant volume discrepancies across thresholds, with wider variations for threshold above 42%. In their analyses no base line value was available, and the conclusion was that WKP volume decreased with time. However, when we analyzed the CT based anatomical volume changes from a baseline volume it was observed a rapid initial increase in volume followed by a decrease; after 7.d.p.i. the volume is again equal the baseline volume. As mentioned, this indicate that the volume change is caused by the amino acid infusion. Together with the study by Sandström et al. it is obvious that volume change occur and might influence dosimetry if not corrected for it.

In this study, we observed anatomical kidney changes based on post-therapy SPECT/CT images of NETs patients treated with ^177^Lu-DOTATATE treatment, providing insights into these physical changes and their implications for kidney dosimetry. In contrast to functional volume, which can vary significantly due to changes in kidney function or radiotracer uptake over time, anatomical volume refers to the actual physical size of the kidney, irrespective of its functional state. Anatomical changes can result from factors such as edema, inflammation, or other physiological responses to treatment. Therefore, understanding of these changes is crucial for an accurate dosimetry.

The kidney absorbed dose estimation based on the WKP defined on single time point CT and used on serial SPECTs acquired at several time points post-treatment could be challenging and has certain limitations. For example, mismatch between SPECT and CT images can affect accuracy of kidney absorbed doses estimate. A major source of this image mismatch such as respiratory and cardiac motion that can make dosimetry challenging by causing artifacts or blurred organ boundaries. However, correct patient positioning during sequential SPECT/CT scans, respiratory gating, and motion correction with appropriate compensation can be possible solutions; if properly implemented it can improve SPECT quantitative accuracy [21, 22]. Further study is required to evaluate the impact of mismatched CT and SPECT images on WKP volume definition amongst sequential SPECT/CT images.

A fixed mean value of the RC, i.e., 0.85 as recommended by the EANM guidelines [23] can be used to achieve sufficient accuracy in kidney dosimetry. It is characterized in the study [18], that the patient-specific RC measured using Monte Carlo Simulation depended on the volume and shape of the WKP parenchyma as well as reconstructed method. However, since RC equal to 0.85 is not patient-specific, it will increase the uncertainty. In the present study we determined an 4% uncertainty increase compared with individualized RC as we used in the reference method for the kidney absorbed dose estimate.

Dosimetry based on manually delineated kidney parenchyma on CT images considers volume changes during therapy and is considered as the ground truth method for kidney dosimetry of radionuclide targeted therapy. However, manually delineating kidneys on CT images acquired at every time point is time consuming. Obviously, kidney dosimetry based on CT images from a single time point is much faster than kidney dosimetry based on CT images from multiple time points. Nevertheless, the fast progress of AI based segmentation tools will make the delineation less time consuming and have the potential to enable effective and accurate dosimetry by time specific WKP volumes [24].

The integration of molecular (SPECT and PET) and anatomical (CT and MRI) imaging techniques enhanced the diagnosis potential. However, mismatch of dual modalities images can alter the true quantitative analysis on molecular imaging. Apparently, SPECT and CT images are acquired sequentially with the patient remaining in the same position while the bed moves between the fields of view to be acquired. In particular, the issues of patient motion and spatial misalignment of the SPECT and CT modalities, data corrections (such as CT-based for photon attenuation scatter corrections), and the choice of CT acquisition protocol in relation to radiation exposure are possible limitations in this study for precise quantitative SPECTs measurement.

## Conclusions

The significant changes in kidney volume over time (from D0 to D7) seem to be in large part due to the intravenous administration of amino acid solutions recommended for renal protection for ^177^Lu-DOTATATE treatment. The WKP change during treatment might introduce an overestimate of the absorbed dose when a single delineated WKP is used for all time points in the determination of the mean activity concentrations. In addition, due to large individual WKP volumes variation, it is advisable to use patient specific RC for minimizing the uncertainties in the absorbed dose calculations.

## Data Availability

The datasets used and analyzed during the current study are available from the corresponding author on reasonable request.
